# Impact of Resilience-Based Exercise on Quality of Life and Cardiac Function in Elderly CHF Patients

**DOI:** 10.1093/geroni/igaf122.2426

**Published:** 2025-12-31

**Authors:** Yanping Zhai

**Affiliations:** Shanxi Bethune Hospital, Taiyuan, Shanxi, China (People’s Republic)

## Abstract

**Objective:**

This study aims to evaluate the effects of a personalized exercise intervention, grounded in psychological resilience theory, on the quality of life and cardiac function of elderly patients with chronic heart failure (CHF). By enhancing psychological resilience, the intervention encourages active participation in exercise, thus improving overall health outcomes.

**Methods:**

In this randomized controlled trial, 80 elderly patients (aged 65 years and older) with chronic heart failure were randomly assigned to either the experimental group or the control group. The experimental group underwent a 12-week personalized exercise program, which included aerobic exercise, strength training, and psychological resilience training, while the control group received standard care. Pre- and post-intervention assessments included the SF-36 quality of life scale, cardiac function evaluations (6-minute walk test, NYHA classification, and left ventricular ejection fraction), and psychological resilience (measured using the CD-RISC scale).

**Results:**

The experimental group demonstrated significant improvements in the 6-minute walk test, left ventricular ejection fraction, and quality of life compared to the control group (P < 0.05). Psychological resilience scores significantly increased post-intervention (P < 0.05), with a positive correlation between resilience improvements and cardiac function.

**Conclusion:**

The personalized exercise intervention based on psychological resilience theory significantly improves cardiac function, quality of life, and psychological resilience in elderly patients with chronic heart failure. These findings suggest that incorporating psychological resilience strategies into treatment plans could enhance overall health and quality of life for heart failure patients.

